# The isoform A of reticulon-4 (Nogo-A) in cerebrospinal fluid of primary brain tumor patients: influencing factors

**DOI:** 10.18632/oncotarget.25278

**Published:** 2018-05-18

**Authors:** Olga Martyna Koper, Joanna Kamińska, Anna Milewska, Karol Sawicki, Zenon Mariak, Halina Kemona, Joanna Matowicka-Karna

**Affiliations:** ^1^ Department of Clinical Laboratory Diagnostics, Medical University of Bialystok, Bialystok, Poland; ^2^ Department of Statistics and Medical Informatics, Medical University of Bialystok, Bialystok, Poland; ^3^ Department of Neurosurgery, Clinical Hospital of the Medical University of Bialystok, Bialystok, Poland

**Keywords:** cerebrospinal fluid, isoform A of reticulon-4 (Nogo-A), primary brain tumor

## Abstract

**Background:**

The influence of isoform A of reticulon-4 (Nogo-A), also known as neurite outgrowth inhibitor, on primary brain tumor development was reported. Therefore the aim was the evaluation of Nogo-A concentrations in cerebrospinal fluid (CSF) and serum of brain tumor patients compared with non-tumoral individuals.

**Results:**

All serum results, except for two cases, obtained both in brain tumors and non-tumoral individuals, were below the lower limit of ELISA detection. Cerebrospinal fluid Nogo-A concentrations were significantly lower in primary brain tumor patients compared to non-tumoral individuals. The univariate linear regression analysis found that if white blood cell count increases by 1 × 10^3^/μL, the mean cerebrospinal fluid Nogo-A concentration value decreases 1.12 times. In the model of multiple linear regression analysis predictor variables influencing cerebrospinal fluid Nogo-A concentrations included: diagnosis, sex, and sodium level. The mean cerebrospinal fluid Nogo-A concentration value was 1.9 times higher for women in comparison to men. In the astrocytic brain tumor group higher sodium level occurs with lower cerebrospinal fluid Nogo-A concentrations. We found the opposite situation in non-tumoral individuals.

**Conclusions:**

Univariate linear regression analysis revealed, that cerebrospinal fluid Nogo-A concentrations change in relation to white blood cell count. In the created model of multiple linear regression analysis we found, that within predictor variables influencing CSF Nogo-A concentrations were diagnosis, sex, and sodium level. Results may be relevant to the search for cerebrospinal fluid biomarkers and potential therapeutic targets in primary brain tumor patients.

**Materials and methods:**

Nogo-A concentrations were tested by means of enzyme-linked immunosorbent assay (ELISA).

## INTRODUCTION

Aside from its role in neurite growth isoform A of reticulon-4 (Nogo-A) has a variety of other functions, e.g. participation in neuronal stem cells differentiation, inhibition of angiogenesis, microglial activity [1].

The role and expression of Nogo-A in brain tumors were studied by several authors [2–7]. *Kuhlman et al.* found strong expression of Nogo-A in 71% of oligodendroglioma, but not in other brain tumors, such as: ependymomas WHO grade II, astrocytoma WHO grade I or II, dysembryoplastic neuroepithelial tumors, clear cell meningiomas, as well as metastases to the brain [4]. The authors suggested Nogo-A expression as a helpful biomarker in distinguishing oligodendrogliomas from astrocytomas WHO grades I and II. Also, the studies of Jin et al. and Marucci et al. revealed the expression of the above-mentioned protein may be useful in the diagnosis of oligodendrogliomas and in differentiating them from other gliomas [5, 6]. Moreover, studies of Jin et al. did not find Nogo-A expression in schwannomas and pituitary adenomas [5]. Additionally, the authors revealed lower migration and invasion abilities of the U87-Nogo-A cells expressing Nogo-A in comparison to control U87MG-E cells not expressing this protein [5]. Some authors indicated Nogo-66, which is an extracellular domain of Nogo-A, as a potential therapeutic factor. Liao et al. found, that Nogo-66 may have inhibiting abilities on the adhesion and migration of human glioma cells *in vitro*. This effect may be mediated *via* the NgR (receptor for Nogo-66), as they revealed that treatment of U87MG cells with NgR antibodies resulted in lowering their potential for adhesion and migration [7].

So far Nogo-A concentrations have been analyzed only in patients serum; however these results were obtained in children with autism spectrum disorders and patients with traumatic brain injury [8–9]. According to best knowledge, there is no data in the available literature regarding the evaluation of Nogo-A concentrations in patients with central nervous system brain tumors in comparison to non-tumoral individuals, neither in patients’ cerebrospinal fluid (CSF), nor in their serum.

In the last few years great effort has been put into searching for diagnostic, prognostic, predictive, and therapeutic response biomarkers in primary brain tumors. Because the search for these kinds of biomarkers still represents an exciting research area, the aim of the current study was to evaluate Nogo-A concentrations in CSF and serum of primary brain tumor patients as compared to non-tumoral individuals.

## RESULTS

We tested Nogo-A concentrations in patients serum, EDTA-K_2_ plasma, and cerebrospinal fluid (CSF) samples. All serum results, obtained both in brain tumors and non-tumoral individuals, were below the lower limit of detection, except in two cases (110 pg/mL and 85 pg/mL, respectively). In EDTA-K_2_ plasma samples all results were below the lower limit of detection. We found Nogo-A results in all cerebrospinal fluid tumors as well as in non-tumoral samples.

**Figure 1:**
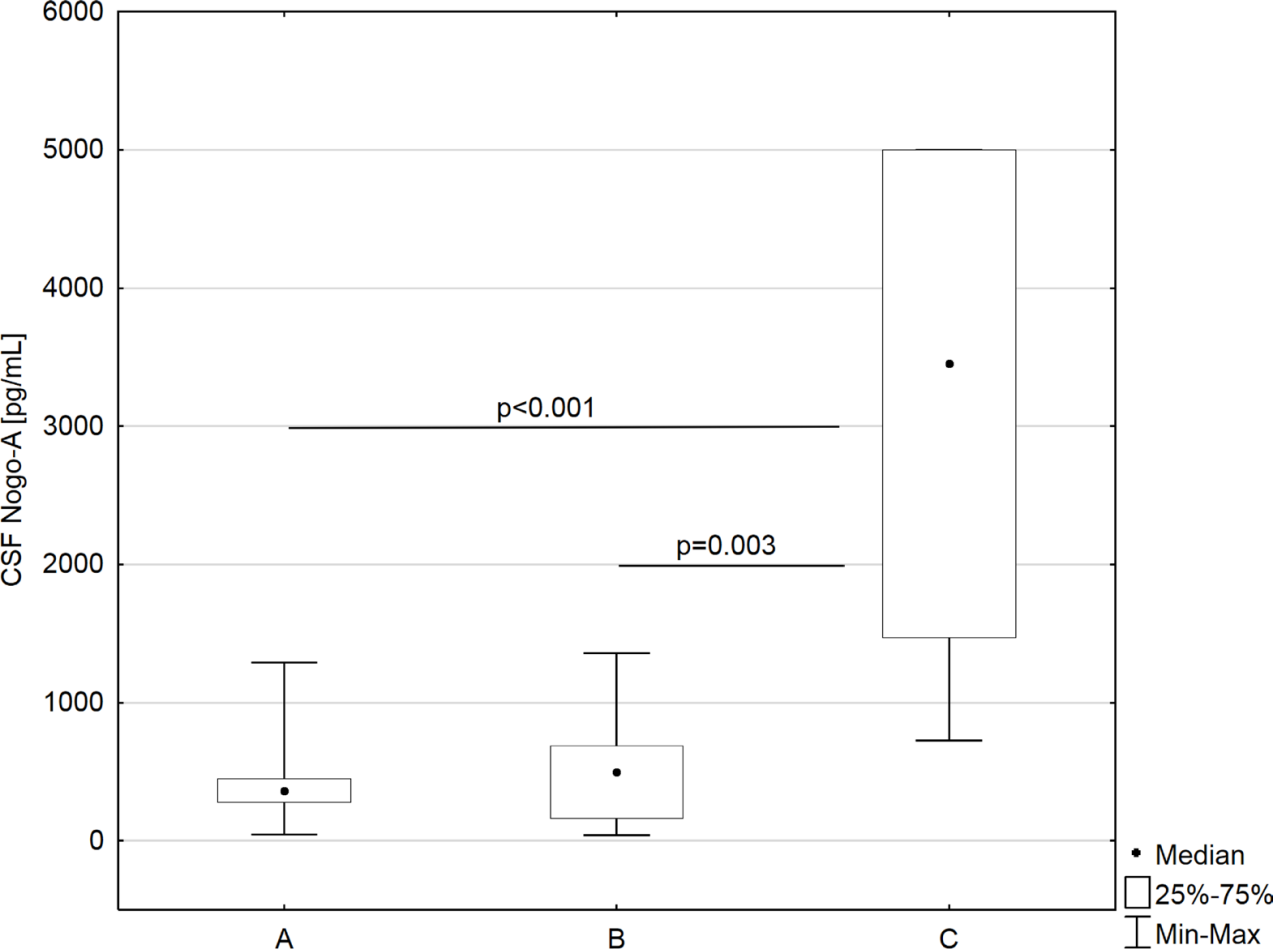
CSF Nogo-A concentrations in astrocytic brain tumors group (**A**), meningeal tumors group (**B**), and non tumoral individuals (**C**).

Median (Me) Nogo-A was a little bit higher in patients with tumors of the meninges (497 pg/mL; IQs:161–687 pg/mL) compared to patients with astrocytic brain tumors (359 pg/mL; IQs:276–449 pg/mL); however this difference was not significant. We also found that CSF Nogo-A concentrations were nearly 10-fold lower in the astrocytic brain tumor group and 7-fold lower in the group of tumors of the meninges compared to non-tumoral subjects (3452 pg/mL; IQs:1471–5000 pg/mL) (*P* < 0.001 and *P* = 0.003, respectively) (Figure 1).

The analysis of the protein tested depending on the sex revealed that females had higher concentrations of Nogo-A (Me = 674 pg/mL; IQs:374–1712 pg/mL) compared to males (Me = 358 pg/mL; IQs:168–893 pg/mL), but these differences were not statistically relevant (*P* > 0.05).

### Correlation coefficient

By means of Spearman's rank method we tested the correlation coefficient; firstly, in the whole study population (tumoral plus non-tumoral individuals), secondly in particular patients subgroups, and finally depending on patients sex.

We found that in the whole study group Nogo-A concentrations negatively correlated with white blood cell count (*R* = −0.407; *P* = 0.010). We did not find any correlation coefficient for Nogo-A in the astrocytic brain tumor subgroup as well as the group of patients with tumors of the meninges. In non-tumoral individuals Nogo-A was positively related to the duration of hospitalization after neurosurgery (*R* = 0.665; *P* = 0.026). We did not find any correlation coefficient for Nogo-A in the male or female group.

### Univariate linear regression analysis

The created model of univariate linear regression analysis adjusted to *R*^2^ = 0.54, which indicates that this model explains 54% of the variance in the dependent variable. We found that if WBC increases by 1 × 10^3^/μL, the mean CSF Nogo-A concentration value decreases 1.12 times (e^β^ = 0.90; *P* = 0.010). For patients with astrocytic brain tumors, the mean CSF Nogo-A concentration value decreases 8.4 times compared to non-tumoral patients (e^β^ = 0.119; *P* < 0.001). For patients with tumors of the meninges, the mean CSF Nogo-A concentration value decreases 8.5 times compared to non-tumoral patients (e^β^ = 0.118; *P* < 0.001) (Table 1).

**Table 1:** Univariate and multivariate linear regression analysis results

		Univariate linear regression analysis		
No	Covariate	β	e^β^ (95% CI)	*P*-value
	Non tumoral diagnosis	base group		
1	Astrocytic brain tumor present	–2.126	0.119 (0.06–0.23)	<0.001
2	Meningeal tumor present	–2.137	0.118 (0.05–0.27)	<0.001
3	WBC [×10^3^/μL]	–0.110	0.90 (0.83–0.97)	0.010
		**Multivariate linear regression analysis**		
**No**	**Covariate**	**β**	**e^β^ (95% CI)**	***P*–value**
	Non tumoral diagnosis	base group		
1	Astrocytic brain tumor present	20.747	1.02^*^10^9^ (6.53–1.61^*^10^17^)	0.032
2	Meningeal tumor present	–1.978	0.14 (0.06–0.31)	<0.001
3	Sex	0.662	1.94 (1.12–3.35)	0.019
4	Na^+^ [mmol/L]	–0.124	1.13 (1.01–1.27)	0.029
5	interaction (Na^+^ [mmol/L]^*^ Astrocytic tumor group)	–0.164	0.85 (0.74–0.97)	0.020

Abbreviations: CI, Confidence Interval; WBC, white blood cells count.

### Multiple linear regression analysis

In the model of multiple linear regression analysis, predictor variables influencing CSF Nogo-A concentrations included: diagnosis, sex, and sodium level. Adjusted *R*^2^ for the created model equals 0.64, which indicates that this model explains 64% of the variance in the dependent variable.

Multiple linear regression analysis revealed that for women the mean CSF Nogo-A concentration was 1.9 times higher for women in comparison to men (e^β^ = 1.939; *P* = 0.019), if other model parameters are fixed. For patients with tumors of the meninges the mean CSF Nogo-A concentration value decreases 7.2 times compared to non-tumoral individuals (e^β^ = 0.138; *P* < 0.001), if other model parameters are fixed (Table 1).

For astrocytic brain tumor patients, we found that if Na^+^ level increases by 1 mmol/L the mean CSF Nogo-A concentration value decreases by 4% (e^β^_4_∙e^β^_5_ = 1.13∙0.85 = 0.96; *P* = 0.029 and *P* = 0.020, respectively), if other model parameters are fixed. For non-tumoral individuals we found that if Na^+^ level increases by 1 mmol/L the mean CSF Nogo-A concentration value increases by 13% (e^β^ = 1.13; *P* = 0.029), if other model parameters are fixed (Table 1).

**Figure 2:**
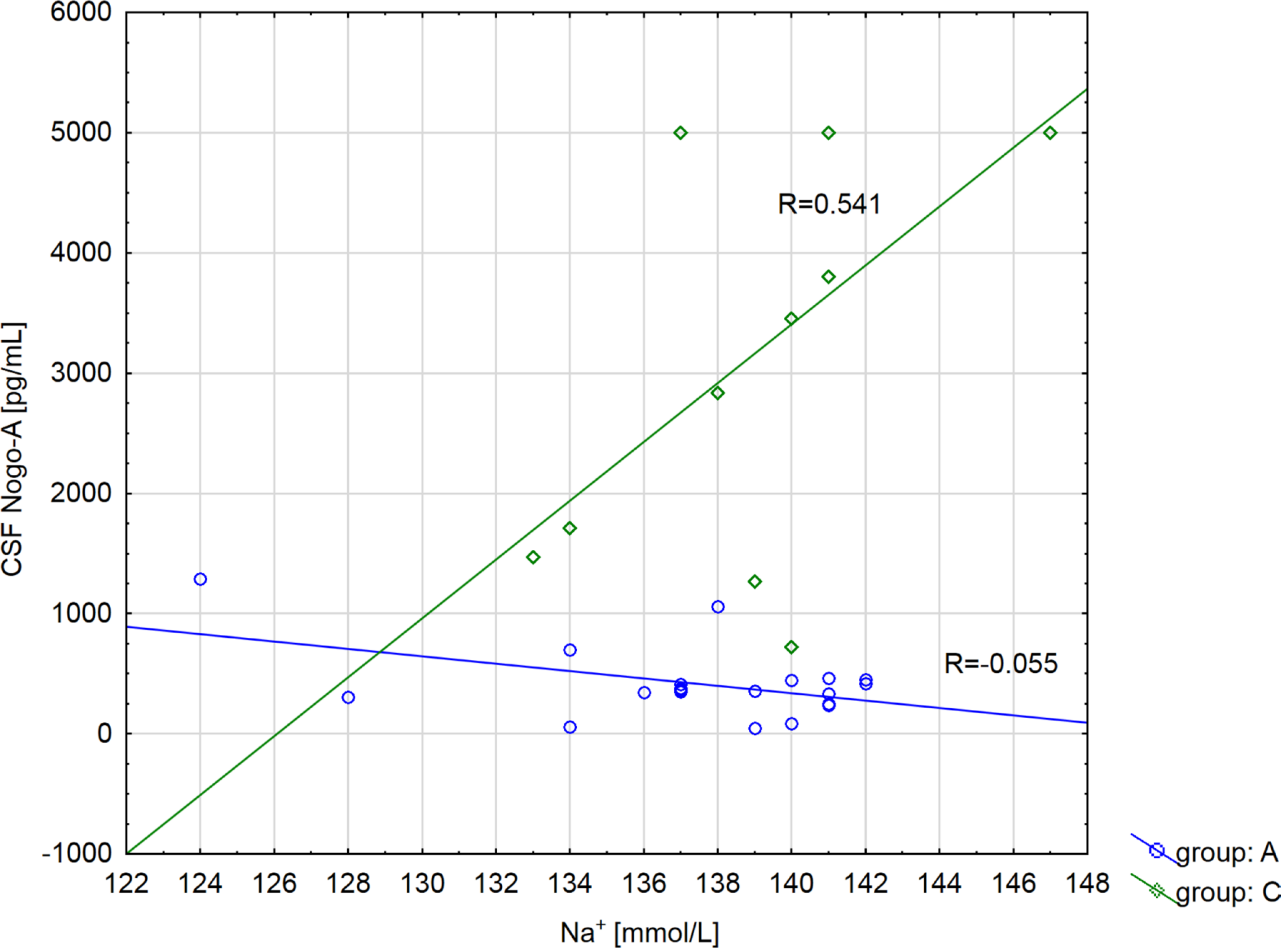
Correlation coefficient of CSF Nogo-A concentration with sodium (Na+) level depending on the diagnosis (A group: astrocytic brain tumors patients; C group: non tumoral individuals)

**Figure 3:**
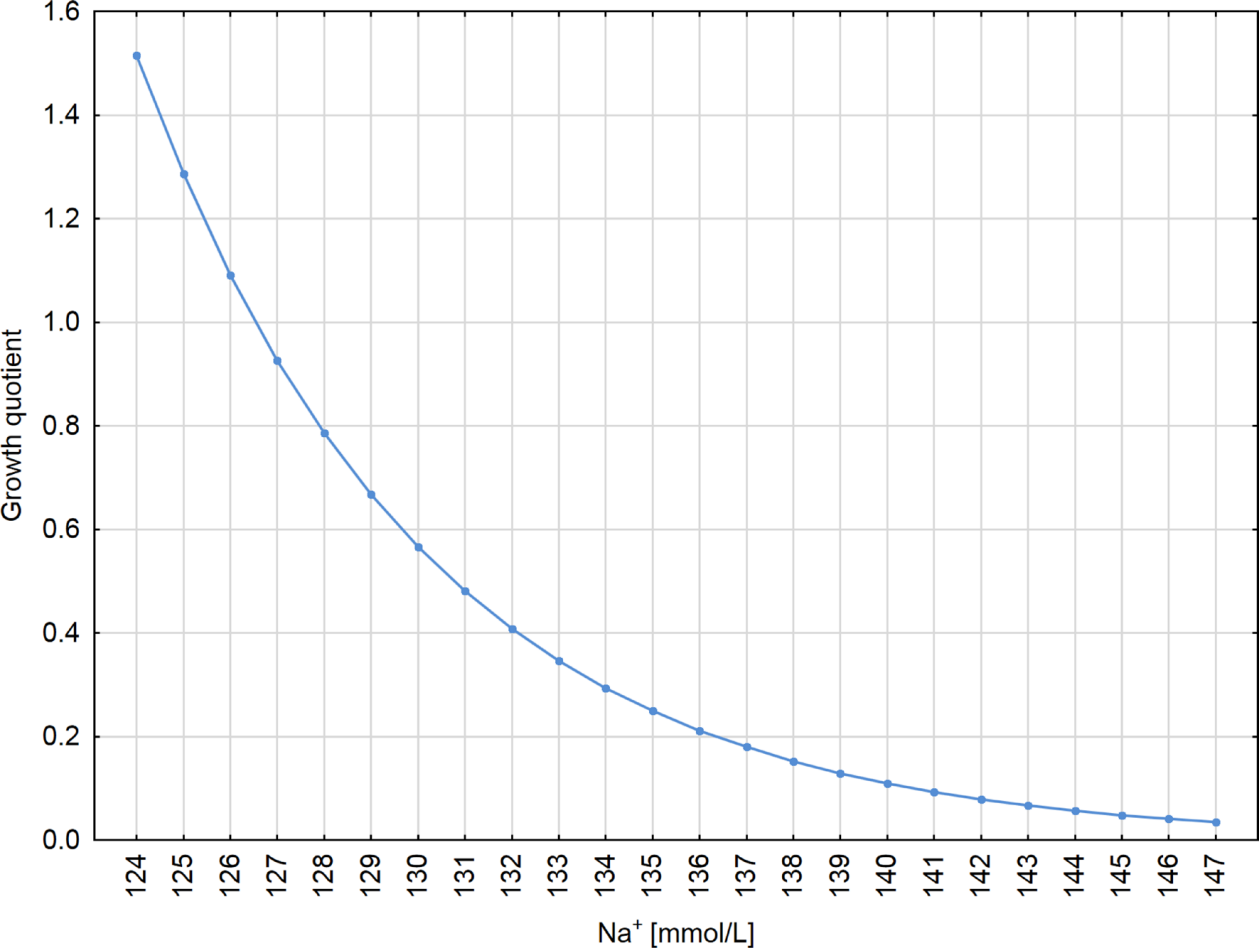
CSF Nogo-A growth quotient for astrocytic brain tumors patients (*vs*. non tumoral individuals) depending on the sodium (Na+) level Please note, that the term “growth quotient” was created for the current study; our purpose was to call the rate on the Y axis, so it is only technical. Such term is not used in the available literature.

For patients with astrocytic brain tumors (*vs*. non-tumoral patients) the mean CSF Nogo-A concentration value changes in relation to sodium (Na^+^) level and increases e^β^_1_∙e^β^_5_∙^Na+^ = e^20.7^∙e^−0.16^∙^Na+^ times (*P* = 0.032 and *P* = 0.020, respectively), if other model parameters are fixed. In the astrocytic brain tumor group, higher sodium levels occur with lower CSF Nogo-A concentrations (*R*^2^ = −0.055). We found the opposite situation in non-tumoral individuals, where a higher sodium levels occur with higher CSF Nogo-A concentrations (*R*^2^ = 0.541) (Figure 2). For example, for astrocytic brain tumor patients with a sodium level that equals 124 mmol/L, the mean CSF Nogo-A concentration value increases 1.52 times *vs*. CSF Nogo-A concentrations in non-tumoral patients with the same sodium level (Figure 3). For astrocytic brain tumor patients with a sodium level that equals 139 mmol/L the mean CSF Nogo-A concentration value decreased 7.7 times (from the result of dividing 1 by 0.13) *vs*. CSF Nogo-A concentrations in non-tumoral patients with the same sodium level (Figure 3).

## DISCUSSION

According to current knowledge, there is no data in the available literature regarding the evaluation of CSF and serum Nogo-A concentrations in patients with astrocytic brain tumors, and moreover in patients with tumors of the meninges. We found that CSF Nogo-A was a little bit higher in patients with meningeal tumors compared to patients with astrocytic brain tumors; however this difference was not statistically relevant. Neither patients serum, nor EDTA samples, are appropriate materials for the evaluation of the above-mentioned protein.

Some authors report Nogo-A expression as a diagnostic tool in differentiating oligodendrogliomas from other gliomas [4, 5]. Additionally, a negative correlation of Nogo-A with oligodendrogliomas malignancy was found [3]. Studies of Jin *et al*. of [5] revealed that Nogo-A decreases malignant glioma cells motility and invasiveness, and thus they suggest a relationship between Nogo-A expression and tumor grade.

Our results showed higher concentrations of CSF Nogo-A in patients with tumors of the meninges, which are mostly low WHO grades [11], as compared to patients with astrocytic brain tumors with higher WHO grades [11]; but the obtained results were not statistically significant, which probably resulted from the small number of meningeal tumor cases. However, Buss et al. [12] revealed that Nogo-A is absent in cerebral blood vessels and the meninges of mature adult brains, which indicates that the source of CSF Nogo-A concentrations in patients with tumors of the meninges is not clear and needs further investigation.

An inflammatory state, which accompanies neoplasms, is rather not the source of CSF Nogo-A in patients with primary brain tumors, as Jurewicz et al. [13] revealed negative Nogo-A Western-blot results in the cerebrospinal fluid of patients with meningo-encephalomyelitis, Creuzfeldt-Jakob disease, systemic lupus erythematosus, neurosarcoidosis, as well as other inflammatory diseases of the central nervous system. The absence of Nogo-A immunoreactivity was also found in the brain tissue of these patients [13].

Our study revealed that in the astrocytic brain tumor group, composed mostly of glioblastoma individuals, a higher sodium level occurs with lower CSF Nogo-A concentrations. This finding may be explained by the fact that glioblastoma cells have a higher expression of Na^+^/K^+^ pump responsible for maintenance of high extracellular Na^+^ concentrations [14–16].

By means of multiple linear regression analysis we found that women had higher levels of CSF Nogo-A compared to men, therefore the evaluation of the above-mentioned protein should depend on a patient's sex.

We also revealed that CSF Nogo-A concentrations were lower in patients with CNS tumors compared to non-tumoral subjects. We hypothesize that lower concentrations of Nogo-A may be related to tumorigenicity, as this protein is responsible for impaired motility and the invasiveness of brain tumor cells line [5]. Moreover, studies of Yan et al. [17] revealed that Nogo-A can modulate immune response within the CNS via the receptor NgR, highly expressed on microglia. The authors revealed, that microglia treated with Nogo-66, one of the Nogo-A's ligand domains, migrate less compared to controls [17].

At this point, we feel obligated to underline, that in our study control subjects were patients with an unruptured intracranial aneurysm, with no history of malignancy. There are two reasons justifying our choice: 1) Lumbar puncture is not routine in the diagnosis of patients with CNS tumors, thus the Local Bioethics Committee gave permission for CSF collection only within neurosurgery; 2) To compare CSF Nogo-A concentrations between tumoral and non-tumoral individuals, cerebrospinal fluid should be collected from the same CNS interspace; so our samples were relatively homogenous.

However, control subjects are definitely non-tumoral individuals and there is a possibility that our choice was not appropriate, as it was reported that Nogo Δ-20, a second Nogo-A ligand domain, disorganizes F-actin filaments structures in multinucleated vascular endothelial cells (MVCEs) as well as MVCE's lamellipodia and filopodia retraction [18]. Nevertheless, this role of Nogo-A should be investigated further.

The first study limitation is the small number of primary brain tumor patients included in the study, which resulted from the difficulties of the cerebrospinal fluid collection procedure (CSF collection had to be performed under a general anesthetic during neurosurgery). Also, CSF sampling in “healthy” control patients is rare nowadays in neuro departments, therefore the collection of these samples took us nearly 2 years. Due to the small number of cases which we were able to include in the study, we did not perform *a priori* (prospective) power analysis, which is generally carried out in a big cooperative studies including hundreds of subjects, especially if a new diagnostic/therapeutic method is to be proposed in the conclusion of a study. Our purpose was far more modest—we only aimed at demonstrating that CSF concentration of Nogo-A is diminished in patients with primary brain tumors and to explore factors which might influence this phenomenon.

The second study limitation is a lack of correlation for Nogo-A concentrations in CSF with their expression in tumor samples, taking into consideration that no report on this is found in the available literature. As a matter of fact we have designed such a study, also including the expression a receptor for Nogo-A and we are currently in the process of applying for funds. Additionally we would like to analyze the expression for NgR, which is a receptor for Nogo-A. Nevertheless, we do not think it is good to wait indefinitely for these “full” results and to not attempt to publish our somewhat preliminary results.

To conclude, cerebrospinal fluid is the best material in which Nogo-A concentrations are detectable by means of ELISA. Neither serum, nor EDTA-K2 plasma are useful for the quantitative evaluation of Nogo-A. Univariate linear regression analysis revealed that cerebrospinal fluid Nogo-A concentrations change in relation to the white blood cell count. Median cerebrospinal Nogo-A concentration in females was higher compared to males, but simple comparison analysis did not reveal a statistical difference. However, in the created model of multiple linear regression analysis we found that among the predictor variables influencing CSF Nogo-A concentrations were diagnosis, sex, and sodium level, as the mean CSF Nogo-A concentration in astrocytic brain tumor patients *vs*. non-tumoral subjects increases for women in comparison to men and changes in relation to sodium levels.

The results of our study may be relevant to the search for CSF biomarkers and/or future therapeutic targets in primary brain tumor patients, however these are only preliminary and should be treated as a foundation for future paths of investigation.

## MATERIALS AND METHODS

### Subjects

The study group consisted of 28 patients with previously untreated primary central nervous system (CNS) tumors, divided into: “A” patients subgroup with astrocytic brain tumors (11 males/9 females; mean age 57 ± years, range 39–73 years) and “B” patients subgroup with meningeal tumors (1 male/7 females; mean age 54 ± years, range 36–68 years).

Table 2 presents the histopathological examination results, WHO grading, gender, and age of particular CNS tumor subjects included in the study. The exclusion criterion was a brain tumor remission in medical history.

**Table 2:** Histopathological examination results, WHO grading, gender, and age of particular CNS tumors subjects included to the study

No.	Histopathological examination	WHO	Gender	Age [years]
Astrocytic brain tumor patients				
1	Diffuse astrocytoma	2	M	39
2	Glioblastoma	4	M	45
3	Glioblastoma	4	F	67
4	Anaplastic astrocytoma	4	M	60
5	Diffuse astrocytoma	2	F	70
6	Glioblastoma	4	M	41
7	Glioblastoma	4	F	73
8	Glioblastoma	4	F	72
9	Gliosarcoma	4	M	58
10	Glioblastoma	4	M	55
11	Anaplastic glioma	3	M	57
12	Glioblastoma	4	F	44
13	Glioblastoma	4	M	57
14	Glioblastoma	4	F	62
15	Pilocytic astrocytoma	1	M	42
16	Glioma	4	F	65
17	Glioblastoma	4	M	59
18	Glioblastoma	4	F	51
19	Glioblastoma	4	M	72
20	Anaplastic astrocytoma	3	F	40
**Meningeal tumor patients**				
1	Transitional meningioma with psammoma bodies	1	F	68
2	Psammomatous meningioma	1	F	45
3	Transitional meningioma	1	F	46
4	Meningothelial meningioma	1	F	47
5	Fibroblastic meningioma	1	F	62
6	Psammomatous meningioma	1	F	72
7	Meningothelial meningioma	1	F	36
8	Anaplastic meningioma	3	M	58

Abbreviations: WHO, World Health Organization; M, male; F, female.

The comparative group was composed of 11 non-tumoral subjects (4 males/7 females; mean age 57 ± years, range 33–70 years) with unruptured intracranial aneurysms, which is usually asymptomatic and only discovered incidentally [10]. The exclusion criteria were cancer in medical history or acute and chronic inflammatory conditions.

Statistical analysis revealed, that patients subgroups were age-matched (*P* > 0.05). The laboratory parameters on admission to the hospital of all subgroups studied are presented in Table 3.

**Table 3:** Laboratory parameters of CNS brain tumors subgroups and non tumoral subjects

	Astrocytic brain tumors (A)	Tumors of the meninges (B)	Non tumoral group (C)	A *vs*. B A *vs*. C B *vs*. C
**WBC [×10^3^/μL]**	12.34 (7.71–17.36)	8.77 (7.10–13.70)	7.33 (5.31–9.30)	NS 0.011 NS
**Na+ [mmol/L]**	139 (137–141)	137 (137–138)	140 (137–141)	NS NS NS
**K+ [mmol/L]**	4.50 (4.21–4.78)	4.17 (4.06–4.56)	4.14 (3.85–4.47)	NS NS NS
**Glucose [mg/dL]**	109 (84–154)	98 (81–124)	96 (90–112)	NS NS NS
**Urea [mg/dL]**	41 (36–58)	30 (22–51)	31 (26–43)	NS NS NS
**Creatinine [mg/dL]**	0.78 (0.76–0.90)	0.70 (0.66–0.76)	0.80 (0.65–0.92)	NS NS NS
**eGFR [mL/min/1.73 m^2^]**	99 (71–112)	95 (82–110)	93 (82–100)	NS NS NS

Values a re present as median and interquartile range. Conversion factors to SI units are as follows: for WBC: 1.0; for glucose: 0.0555; for urea: 0.357; for creatinine: 88.402.

The study was conducted in agreement with the Helsinki-II-declaration and was approved by the Bioethics Human Research Committee of the Medical University of Bialystok (Permission No. R-I-002/383/2015). All subjects included in the study gave their written informed consent.

### Sample collection and storage

Procedures on patients were performed under a general anesthetic during neurosurgery at the Department of Neurosurgery at the Clinical Medical Hospital in Bialystok. Craniotomy was performed on all subjects included in the study. In patients undergoing tumor resection its size and localization was tailored according to location and size of the tumor, preferred access route and surrounding anatomy. Clipping of an aneurysm required an opening in the fronto-temporal region (aka pterional craniotomy). Regardless of operation type or location, after placing the patient's head in a three-pin Mayfield headholder the surgical field was prepared in the standard fashion. Skin incision preceded lifting of bone flap and lancing of dura mater, which allowed for a visualization of arachnoid membrane and subarachnoid space. With the aid of an operating microscope the subarachnoid space was carefully opened and about 2–3 mL of CSF aspirated with a single-use, sterile syringe and soft venous catheter. The aforementioned steps were taken in the very beginning of each procedure, before any potential bleeding occurred. This routine allowed for keeping the CSF clean of blood and not mixed with warm saline solution used as an irrigation.

Blood collected in tubes without anticoagulant, in EDTA-K2 tubes, and CSF samples were centrifuged for 20 minutes at 1000 × g. The obtained serum, plasma, and CSF supernatant were stored at –80° C until further analysis.

### Human RTN4 (Reticulon 4) concentrations analysis

Concentrations of Nogo-A were measured using Human RTN4 ELISA (enzyme-linked immunosorbent assay) kit (Catalogue No.: EH3732; Wuhan Fine Biological Technology Co., Ltd.) according the manufacturer's instruction. Samples were not diluted prior to analysis. According to the Vendor's instruction the range for the detection of Human RTN4 is between 78.125–5000 pg/mL; sensitivity of the assay kit is < 46.875 pg/mL. Between zero well (Standard dilution buffer) and the first standard (78.125 pg/mL) some optic density (OD) absorbance may be read and transformed into Nogo-A concentration. For example the Vendor of the assay kit referred to the value of 0.112 of optic density (OD) at 450 nm for the zero well and the value of 0.242 OD at 450 nm for the 78.125 pg/mL standard value. In our study for brain tumor cerebrospinal fluid samples we found three values, which were below 78 pg/mL (42 pg/mL, 47 pg/mL, and 65 pg/mL) and one of them was below the sensitivity of the kit referred to by the Vendor (< 46.875 pg/mL).

### Statistical analysis

The obtained results were statistically analyzed with the use of the STATISTICA 12.0 PL software (StatSoft Inc., Tulsa, USA) and STATA 12.1 (StataCorp LP). The concentrations of parameters tested did not follow a normal distribution in the preliminary statistical analysis (Shapiro-Wilk test), thus nonparametric statistical analysis was employed. The Mann–Whitney test was used in order to compare two independent samples, and the Kruskal-Wallis test was used for the comparison of three samples. Correlation coefficients were obtained by applying Spearman's rank method. If not otherwise stated, the values for each given measured variable are given as medians and interquartile ranges. Univariate linear regression analysis as well as multiple linear regression analysis models were created. Differences were considered statistically significant for *P* < 0.05.

## Abbreviations

CNS: central nervous system
CSF: cerebrospinal fluid
EDTA-K_2_: ethylenediaminetetraacetic acid dipotassium
ELISA: enzyme-linked immunosorbent assay
MVCEs: multinucleated vascular endothelial cells
NgR: receptor for Nogo-A
Nogo-A: isoform A of reticulon-4
WBC: white blood cells count
WHO: World Health Organization
